# Supplementary material for: Synthesis of Ammonium-Based Ionic Liquids for the Extraction Process of a Natural Pigment (Betanin)
Source: Molecules. 2021 Sep 8;26(18):5458. doi: 10.3390/molecules26185458 (PMC8465181; doi:10.3390/molecules26185458)
Supplement: Supplementary file 1 [file molecules-26-05458-s001.zip › molecules-1314353-supplementary.pdf]

molecules

MDPI

Article

Supplementary Material: Synthesis of Ammonium-Based Ionic Liquids for the Extraction Process of a Natural Pigment (Betanin)

Pedro Morales-García [1], Evelyn Y. Calvillo-Muñoz [1], Irina V. Lijanova [1,*], Natalya V. Likhanova [2], Octavio Olivares-Xometl [3] and Paulina Arellanes-Lozada [3]

Calculation of Extraction tests of betanin

This process was carried out mixing equimolar quantities of $[TBMA]^{+}[HEX]^{-}$ and another IL obtained after the anionic exchange. In the end, eight extraction IL complexes were obtained and used in the first extraction tests. A 105-mg/L solution was prepared using commercial betanins (12% of pigment), deionized water and ascorbic acid adjusted at 0.10% (w/v) in the final solution (final pH 6). Quantities equivalent to 0.001 and 0.60 mmol of extraction complexes were weighed in 4-mL jars; later, 3 mL of pigment solution were added to each jar. The final system was stirred with a magnetic bar for 1 h at environment pressure and temperature. After the stirring process, the remaining betanins were quantified using Equation (1), which was done by making a calibration curve at different concentrations of betanins dissolved in water with 0.10% of ascorbic acid (pH 6). C_f is the final concentration of betanins in mg/L, D_F represents the dilution factor and Abs is the absorbance of the sample at 538 nm.

$$C_f = D_F\left(\frac{Abs - 0.0035}{0.1154}\right) \quad (1)$$

The C_f calculation helped estimate the extraction percentage (P_{ex}), Equation (2). C_i is the pigment concentration at the beginning.

$$P_{ex} = 100 * \left(\frac{C_i - C_f}{C_i}\right) \quad (2)$$

The concentration of betanins in the organic phase (C_o) was determined using Equation (3). V_w and V_o are the volumes of the aqueous and organic phases. The extraction complex with the highest P_{ex} was chosen to be used in other tests with natural red beet juice.

$$C_o = \frac{(C_i - C_f) * V_w}{V_o} \quad (3)$$

The red beet root was chopped in small 5-mm cubes and frozen at -5 °C. Fifty grams were weighed and mixed with a solution of ascorbic acid at 0.10% (w/v) in a porcelain mortar.

The cubes were crushed until obtaining fine pulp, which was filtered using vacuum in order to collect the liquid extract in a flask. By the end of this process, 60 mL of the extract

were recovered and assessed by UV-vis at 538 nm using Equation (1) to calculate the betalaine content in mg/L.

Calculation of Desorption process

Once the aqueous phase was removed, the remaining organic phase was treated with 400 μL of ethanol and 700 μL of sodium acetate solution at specific concentrations; 5, 10, 15 and 20 % (w/v) were tested to determine which of them could provide the best results. The sodium acetate solution was enriched with 0.10% of ascorbic acid. The system was stirred for 2 min and later it was left to rest for 20 min to stabilize the phases. The desorption percentage (P_{des}) was calculated by the application of Equations (4) and (5):

$$C_{TD} = \frac{C_o * V_{oD}}{V_{wD}} \quad (4)$$

$$P_{des} = 100 * \left(\frac{C_{TD} - C_D}{C_{TD}}\right) \quad (5)$$

C_{TD} is the theoretical concentration of betanin in the new aqueous phase after desorption, V_{oD} and V_{wD} are the volumes of the new organic and aqueous phases and C_D is the concentration of the separated pigment from the new aqueous phase, which was determined by means of Equation (1) as well. Finally, the sodium acetate solution that presented the highest P_{des} was used in the desorption process of betalains recovered from natural red beet juice.

Partition coefficient ($K_{LI's/w}$) estimation

This parameter was determined through the models by Betageri, G. V., *et al.*, 1987 [28] (Equations 6 and 7) and Lima, Á. S., *et al.*, 2017 [22] (Equation 8),

$$K^m_{LI's/W} = \frac{W_w(C_i - C_f)}{C_f * W_o} \quad (6)$$

$$K_{LI's/W} = K^m_{LI's/W} * \left(\frac{\rho_o}{\rho_w}\right) \quad (7)$$

$$K_{LI's/W} = \frac{C_o}{C_f} \quad (8)$$

where $K^m_{LI's/W}$ and $K_{LI's/W}$ are the partition coefficients in molality and molarity, respectively; W_w and W_o represent the mass of the aqueous and organic phases, and C_o and C_f refer to the concentration of betanins in the organic and aqueous phases after the extraction process.
